# Clinician Adherence to Hypertension Screening and Care Guidelines

**DOI:** 10.1001/jamanetworkopen.2023.47164

**Published:** 2023-12-12

**Authors:** Nikkil Sudharsanan, Vasanthi Subramonia Pillai, Caterina Favaretti, Jithin Jose, Sandra Jose, Margaret McConnell, Mohammed K. Ali

**Affiliations:** 1 Professorship of Behavioral Science for Disease Prevention and Health Care, TUM School of Medicine and Health, Technical University of Munich, Munich, Germany; 2 Heidelberg Institute of Global Health, Heidelberg University, Heidelberg, Germany; 3 LEAD at Krea University, Chennai, Tamil Nadu, India; 4 Department of Global Health and Population, Harvard T.H. Chan School of Public Health, Harvard University, Cambridge, Massachusetts; 5 Emory Global Diabetes Research Center, Woodruff Health Sciences Center, Emory University, Atlanta, Georgia; 6 Hubert Department of Global Health, Rollins School of Public Health, Emory University, Atlanta, Georgia; 7 Department of Family and Preventive Medicine, School of Medicine, Emory University, Atlanta, Georgia

## Abstract

This quality improvement study assesses opportunistic blood pressure measurement, communication of blood pressure reading to adult patients, and recommendation for a follow-up visit at health care facilities in 2 major cities in India.

## Introduction

Uncontrolled hypertension is a leading risk factor for mortality globally and affects 26% of adults in India.^1^ Underdiagnosis is a primary cause of poor hypertension control as only 37% of Indians with hypertension are diagnosed.^1^ To increase diagnosis, Indian guidelines recommend that clinicians opportunistically screen adults for hypertension at all points of care.^2^ This recommendation has substantial policy potential since Indian adults report frequent health care visits. Underdiagnosis despite guideline recommendations and frequent visits suggests that clinicians are not consistently screening for hypertension, leading to missed opportunities for increasing diagnosis.^3^ While there is evidence of poor guideline adherence in other care domains,^4,5,6^ there is limited research on clinician adherence to hypertension screening guidelines in India.

## Methods

We assessed clinician adherence to hypertension screening guidelines using unannounced standardized patients (SP), individuals who were trained to pose as real patients. For this quality measurement study, SPs sought care for lower back pain (a condition unrelated to hypertension) in 301 randomly sampled primary health facilities in Chennai and Kolkata, 2 major cities in India. The Indian IFMR Human Subjects Committee approved this study and waived informed consent because of the unannounced SP design. We followed the STROBE reporting guideline.

After each visit, SPs reported the clinical actions they received from facility clinicians. Following Indian guidelines,^2^ study outcomes were whether clinicians opportunistically measured blood pressure (BP) at all, measured BP at least twice, communicated measurements to the SPs, and advised a follow-up visit when the measurement was 140/90 mm Hg or higher. Results were presented as the percentage of SP visits in which each outcome occurred overall and stratified by clinic (clinic type, location, and patient load) and patient characteristics (sex and age).

Data analysis was conducted using Stata 15 (StataCorp LLC) and R 4.2.2 (R Project for Statistical Computing). All hypothesis tests were 2-sided; *P* = .05 indicated significance. Additional information is provided in the eAppendix, eMethods, and eFigure in Supplement 1.

## Results

**Figure 1.**
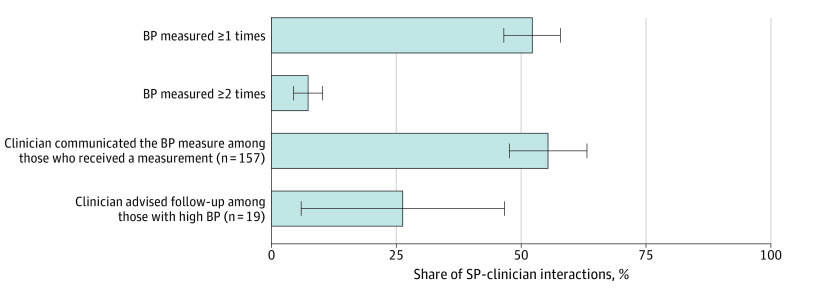
Opportunistic Screening Rates in Chennai and Kolkata, India, in 2022 There were 301 standardized patient (SP)–clinician consultations. Communication was measured only among the 157 consultations during which clinicians measured blood pressure (BP); follow-up advice given was measured only among the 19 consultations during which clinicians measured BP and the measurement was greater than 140/90 mm Hg. Error bars represent 95% CIs.

Eleven SPs (6 females and 5 males; mean [SD] age, 45 [6] years) conducted 301 visits. Clinicians measured BP at least once in 52% (95% CI, 47%-58%) of visits and at least twice in 7% (95% CI, 4%-10%) of visits (Figure 1). The SPs received communication regarding their BP in 55% (95% CI, 48%-63%) of visits in which their BP was measured. There was an elevated BP level in 19 of 157 visits (12.1%) in which BP was measured. Clinicians advised a follow-up visit in 26% (95% CI, 6%-47%) of these visits.

**Figure 2.**
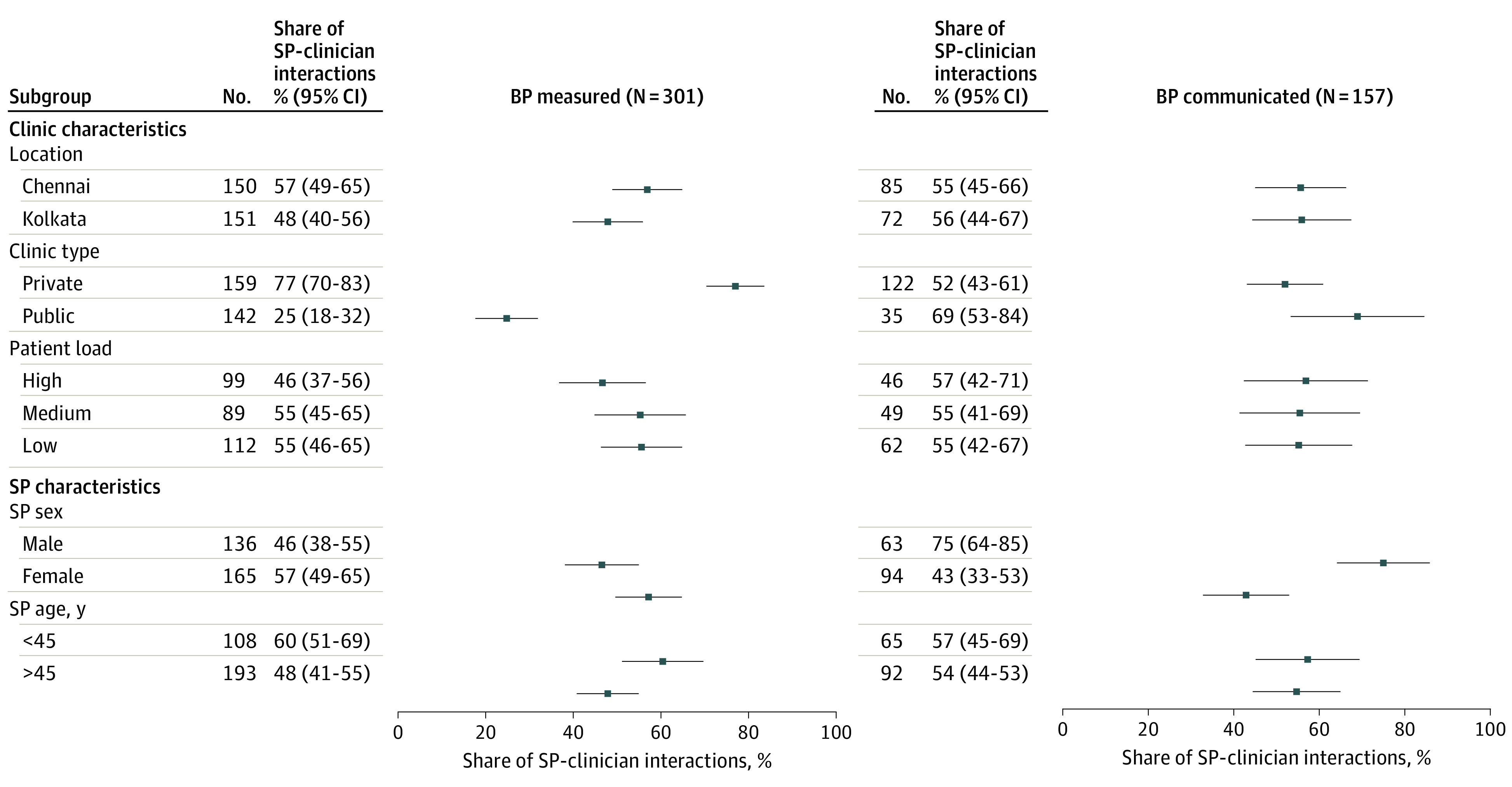
Opportunistic Screening Quality by Clinic and Patient Characteristics in Chennai and Kolkata, India in 2022 Standardized patients (SPs) were between ages 36 and 55 years, with a mean (SD) age of 45 (6) years. Blood pressure (BP) screening and communication rates for SPs above vs below the mean age were compared. The number of patients in the waiting room was used to measure patient load and classify it into high (≥7), medium (≤3 to ≤6), and low (≤2) based on tertiles. Error bars represent 95% CIs.

Clinicians in private vs public facilities were far more likely to measure BP at least once (77% vs 25%; *P* < .001) (Figure 2). Conditional on being measured, males were more likely to receive communication regarding their BP than females (75% vs 43%; *P* < .001). We found no differences across other characteristics or outcomes.

## Discussion

We found low clinician adherence to opportunistic hypertension screening guidelines in Chennai and Kolkata. Clinicians measured BP in approximately only half of consultations with SPs. We also found poor clinician communication. When clinicians measured BP, they communicated results to SPs in only over half of consultations, with less communication provided to females than males.

These results suggest that hypertension is being substantially underdiagnosed in urban India as clinicians frequently skip essential screening actions. Even after BP measurement, awareness among patients could be low due to poor communication by clinicians. This study was limited by use of only 2 urban centers and inability to assess long-term or follow-up care.

The results and the broader literature from India suggest that quality-improvement interventions need to directly target clinician behavior. Commonly used approaches for clinician behavior change, such as financial incentives and sanctions, may be challenging to implement in India due to limited resources, oversight, and regulation. In India, approaches such as clinical support systems, task-shifting, and nonfinancial incentives may be more practical. Changing clinician behavior is crucial to translating India’s ongoing primary care improvement efforts into increased hypertension control.

## Notes

Supplement 1. **eAppendix.** Ethical Considerations **eMethods.** Study Sample **eFigure.** Sampling of Healthcare Facilities, Chennai and Kolkata, 2022

Supplement 2.
Data Sharing Statement
