# Supplementary material for: Clinician Adherence to Hypertension Screening and Care Guidelines
Source: JAMA Netw Open. 2023 Dec 12;6(12):e2347164. doi: 10.1001/jamanetworkopen.2023.47164 (PMC10716733; doi:10.1001/jamanetworkopen.2023.47164)
Supplement: Supplement 1. — eAppendix. Ethical Considerations eMethods. Study Sample eFigure. Sampling of Healthcare Facilities, Chennai and Kolkata, 2022 [file jamanetwopen-e2347164-s001.pdf]

# Supplemental Online Content

Sudharsanan N, Subramonia Pillai V, Favaretti C, et al. Clinician adherence to hypertension screening and care guidelines. *JAMA Netw Open*. 2023;6(12):e2347164. doi:10.1001/jamanetworkopen.2023.47164

**eAppendix.** Ethical Considerations

**eMethods.** Study Sample

**eFigure.** Sampling of Healthcare Facilities, Chennai and Kolkata, 2022

This supplemental material has been provided by the authors to give readers additional information about their work.

© 2023 Sudharsanan N et al. *JAMA Network Open*

**eAppendix. Ethical Considerations**

We took the following steps to minimize the risk to the individuals posing as SPs. First, we chose a case presentation (lower back pain) that does not generally lead to invasive measurements; we also extensively trained SPs on strategies for refusing any invasive measurements should they be suggested. In small communities, there is a risk that SPs will be recognized in the clinic and suffer a reputation loss. We worked in the context of very large cities and thus believe that this risk was significantly reduced.

We took the following steps to minimize the risk to clinicians. We did not identify any specific clinicians or facilities and only presented aggregated averages here. We also did not collect any identifying information on clinicians other than their sex and thus cannot link any of our responses to a specific clinician. We also took steps to minimize the risk to other patients. To avoid crowding care for real patients, we sent only one SP to each facility. We also trained SPs to step away from the clinician in the case of a medical emergency for another patient.

Deception associated with SP studies raises important ethical considerations. Before conducting our study, we received ethical approval from a local Indian ethics board, including a waiver of clinician consent. There is also a substantial precedent for conducting SP studies in India, and we followed the best practices of these prior studies. Ultimately, the study design was justified as it is the only way to objectively measure clinician screening behavior for an important health condition that carries a large population health burden in India.

© 2023 Sudharsanan N et al. *JAMA Network Open*

**eMethods. Study Sample**

SPs visited both public and private primary healthcare settings in both cities. For public facilities, we randomly sampled 50% of all facilities in both cities using publicly published lists taken from the city government websites (69 in Chennai and 73 in Kolkata). Since there are no similar listings of private healthcare facilities, we first created a sampling frame by extracting facility information from JustDial and Practo, two widely used online listing services similar to the YellowPages. We then randomly sampled private facilities from these constructed lists. We selected 81 private facilities in Chennai and 78 in Kolkata to reach a total sample size of 150 facilities per city. eFigure below describes the sampling strategy.

Within selected facilities, SPs either met with just a physician (34%, N=301), just a nurse (8%, N=301), or both (58%, N=301). Since each facility was visited once, we use "clinician" to refer to whomever provided care to the SP during their facility visit.

© 2023 Sudharsanan N et al. *JAMA Network Open*

**eFigure. Sampling of Healthcare Facilities, Chennai and Kolkata, 2022**

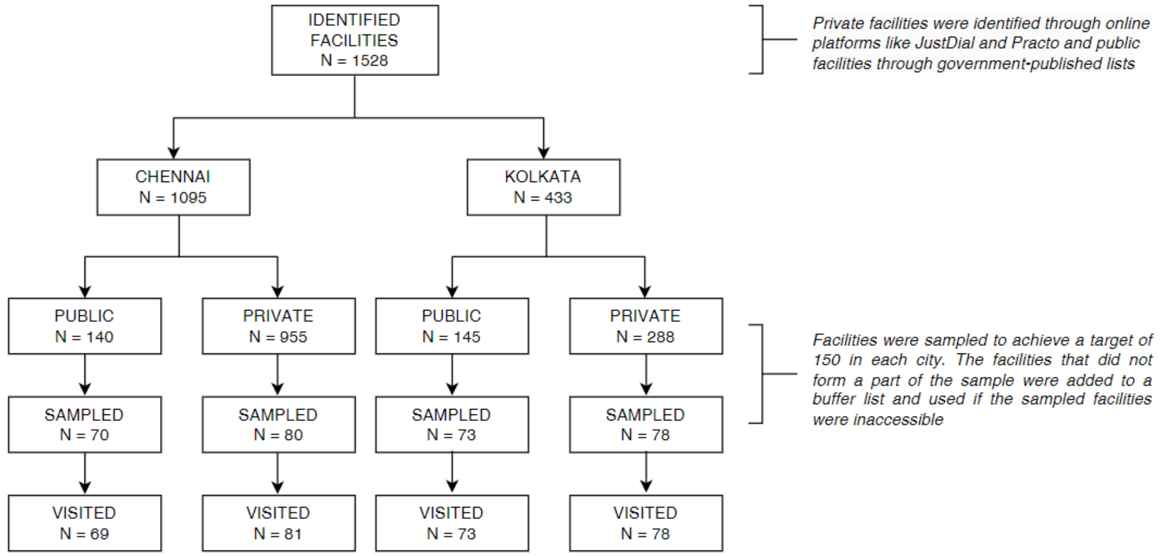


*Recruitment and Training of SPs:* We recruited a total of 11 SPs, with 6 SPs in Chennai and 5 SPs in Kolkata. Among the 11 SPs, 6 were female and 5 were male and the SPs were between 36 and 55 years old. We trained the SPs to provide standardized responses to a range of potential questions and conducted extensive mock sessions before the start of the study to ensure that SPs were comfortable posing as patients and correctly followed the case scripts. We conducted one SP visit per facility for a total sample of 301 SP-clinician consultations (~150 per city). Within one hour of their visit, SPs met with a survey enumerator who debriefed them and collected information about the consultation.

© 2023 Sudharsanan N et al. *JAMA Network Open*
