# Supplementary material for: Clinician Adherence to Hypertension Screening and Care Guidelines
Source: JAMA Netw Open. 2023 Dec 12;6(12):e2347164. doi: 10.1001/jamanetworkopen.2023.47164 (PMC10716733; doi:10.1001/jamanetworkopen.2023.47164)
Supplement: Supplement 2. — Data Sharing Statement [file jamanetwopen-e2347164-s002.pdf]

Data Sharing Statement

Sudharsanan. Clinician Adherence to Hypertension Screening and Care Guidelines. *JAMA Netw Open*. Published December 12, 2023. doi:10.1001/jamanetworkopen.2023.47164

Data
Data available: Yes
Data types: Deidentified participant data
How to access data: nikkil.sudharsanan@tum.de
When available: With publication

Supporting Documents
Document types: None

Additional Information
Who can access the data: anyone requesting data
Types of analyses: for research
Mechanisms of data availability: without investigator support
